# In Silico Study to Predict the Structural and Functional Consequences of SNPs on Biomarkers of Ovarian Cancer (OC) and BPA Exposure-Associated OC

**DOI:** 10.3390/ijms23031725

**Published:** 2022-02-02

**Authors:** Aeman Zahra, Marcia Hall, Jayanta Chatterjee, Cristina Sisu, Emmanouil Karteris

**Affiliations:** 1 Biosciences, College of Health, Medicine and Life Sciences, Brunel University London, Uxbridge UB8 3PH, UK; aeman.zahra@brunel.ac.uk (A.Z.); marcia.hall@nhs.net (M.H.); jayanta.chatterjee1@nhs.net (J.C.); 2 Mount Vernon Cancer Centre, Northwood HA6 2RN, UK; 3 Faculty of Health and Medical Sciences, School of Biosciences and Medicine, University of Surrey, Guildford GU2 7XH, UK

**Keywords:** missense mutations, protein modelling, *SLC4A11*, uterine corpus endometrial carcinoma

## Abstract

Background: Recently, we have shown that seven genes, namely *GBP5*, *IRS2*, *KRT4*, *LINCOO707*, *MRPL55*, *RRS1* and *SLC4A11,* have prognostic power for the overall survival in ovarian cancer (OC). Methods: We present an analysis on the association of these genes with any phenotypes and mutations indicative of involvement in female cancers and predict the structural and functional consequences of those SNPS using in silico tools. Results: These seven genes present with 976 SNPs/mutations that are associated with human cancers, out of which 284 related to female cancers. We have then analysed the mutation impact on amino acid polarity, charge and water affinity, leading to the identification of 30 mutations in gynaecological cancers where amino acid (aa) changes lead to opposite polarity, charges and water affinity. Out of these 30 mutations identified, only a missense mutation (i.e., R831C/R804C in uterine corpus endometrial carcinomas, UCEC) was suggestive of structural damage on the *SLC4A11* protein. Conclusions: We demonstrate that the R831C/R804C mutation is deleterious and the predicted ΔΔG values suggest that the mutation reduces the stability of the protein. Future in vitro studies should provide further insight into the role of this transporter protein in UCEC.

## 1. Introduction

Ovarian carcinoma (OC) is the most fatal gynaecologic malignancy, accounting for more than 200,000 deaths annually (WHO; Cancer Today). Over 80% of patients with advanced OC will relapse, and despite further good remissions from additional chemotherapy and surgery, they will usually die from their disease [1]. The median progression-free survival (PFS) for relapsed ovarian cancer (ROC) patients who last had treatment within 3–12 months previously is 4–9 months, with overall survival (OS) of ~12–20 months [2]. It should be noted that there is a genetic variation of response to chemotherapy and subsequently to tumour progression [3].

A plethora of studies—primarily via genome-wide association studies—have conclusively demonstrated an association between single-nucleotide polymorphisms (SNPs) and cancer risk [4]. There is a high frequency of SNPs occurrence in the human genome. In particular, amino acid point mutations or non-synonymous single-nucleotide polymorphisms (nsSNPs) may alter the structure and subsequently affect the function of the mutated protein [5]. More than 13,000 known SNPs are in exon regions, of which 58% are nsSNPs [6]. Indeed, a number of nsSNPs are associated with an increased cancer risk [7]. For example, nsSNPs in codon 31 of the *p21* gene are associated with an increased risk of cervical cancer development [8].

Apart from genetic changes, exposure to endocrine-disrupting chemicals (EDCs) can disturb the normal functions of the endocrine system in humans and increase the risk of adverse health effects [1]. Bisphenol A (BPA) (an EDC) has a pro-carcinogenic impact in hormone-dependent and hormone-independent cancers [9,10,11]. BPA exposure is reported to alter the cancer cells’ biological behaviours, particularly, proliferation, invasion, growth, survival, migration and apoptosis [9,12,13,14,15,16]. Recently, we have identified seven genes that have prognostic power for the overall survival in OC, namely Guanylate Binding Protein 5 (*GBP5*), Insulin Receptor Substrate 2 (*IRS2*), Keratin 4 (*KRT4*), long intergenic non-protein coding RNA 707 (*LINC00707*), Mitochondrial Ribosomal Protein L55 (*MRPL55*)*,* Ribosome Biogenesis Regulator 1 Homolog (*RRS1*) and Solute Carrier Family 4 Member 11 (*SLC4A11*). Out of these seven genes, *KRT4* appears to be a biomarker of BPA exposure-associated OC, whereas *GBP5*, *LINC00707* and *SLC4A11* appear to be biomarkers of disease [17].

In this study, we aimed to predict the structural and functional consequences of SNPs mapped in genetic variants of these seven biomarkers in gynaecological malignancies.

## 2. Results

### 2.1. Landscape of Mutations in Seven Biomarker Genes Based on TCGA, cBioPortal and UK Biobank

**Figure 1:**
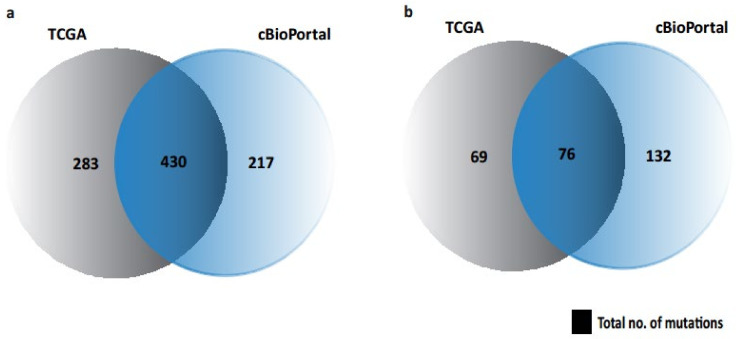
Venn diagram showing the possible mutations/SNPs associated with seven biomarkers in cBioPortal and UCSC Xena repository. (**a**) Mutations in human cancers. (**b**) Mutations in female cancers.

We have previously identified seven biomarkers of OC and exposure-associated OC, as discussed [17]. We found that these 7 biomarkers represent 976 and 284 SNPs/mutations associated with human cancers and female cancers, respectively. It should be noted that in Figure 1, we did not illustrate UK BioBank (PhenoScanner)-associated mutations (Table 1) as it has no overlapping/intersection with any other database (cBioPortal or TCGA).

**Figure 2:**
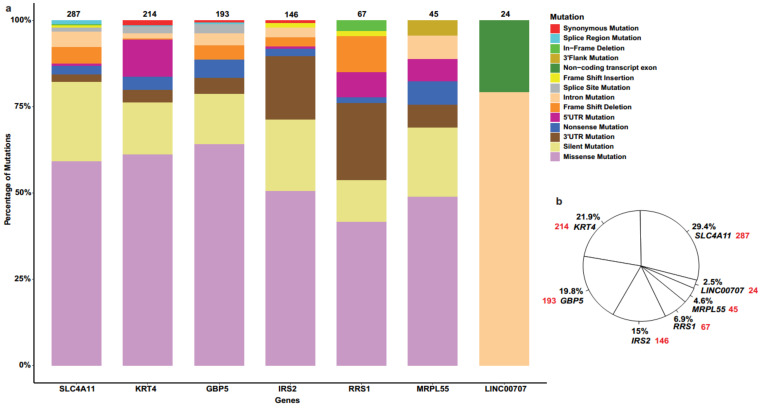
(**a**) Bar plot representing types of SNPs/mutations associated with seven biomarkers in human cancers. (**b**) Pie chart demonstrating the percentage distribution of 976 SNPs for 7 biomarkers in human cancers, where red colour represents the number of mutations in each gene.

**Figure 3:**
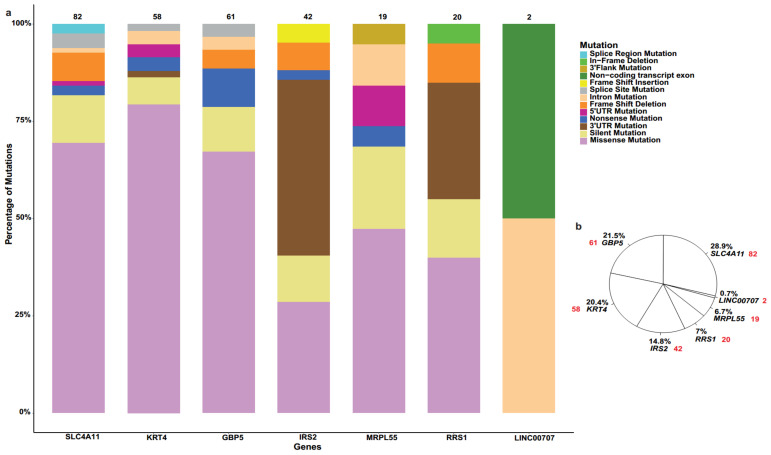
(**a**) Bar plot indicating different types of mutations associated with seven biomarkers in female cancers. (**b**) Pie chart specifying the percentage distribution of 284 SNPs for 7 biomarkers in female cancers, where red colour represents the number of mutations in each gene.

These SNPs were further analysed according to the number and percentage of mutations associated with seven biomarkers of interest in human cancers (Figure 2) and female cancers (Figure 3), along with mutation types.

**Figure 4:**
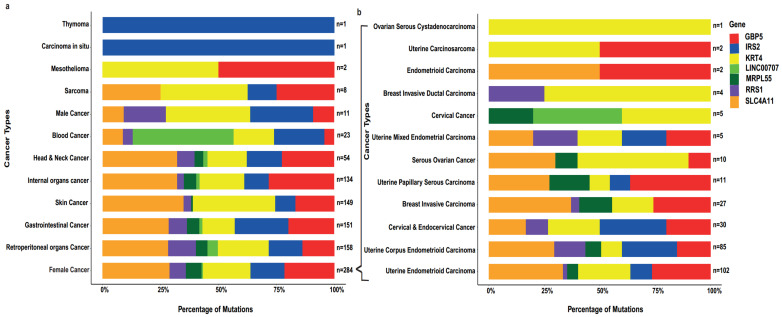
(**a**) Bar plot showing the sample size and percentage of mutation in seven biomarkers in each human cancer type, (**b**) with emphasis on female cancers.

Further, we analysed the percentage of mutation and sample size in all related human cancers (Figure 4a) and female cancers (Figure 4b), along with associated biomarkers (highlighted in seven colours). Table 2 summarises the mutation impact on protein structure and function, including amino acid (aa) polarity, charges and water affinity.

**Figure 5:**
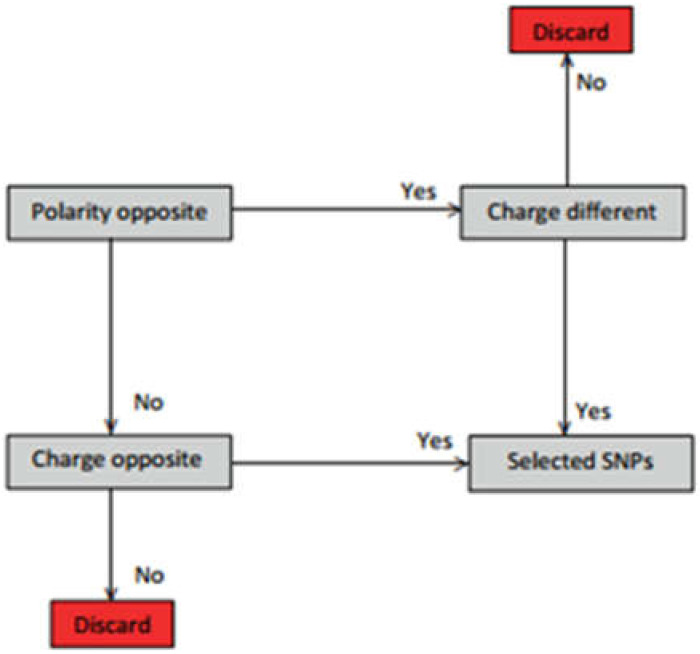
Amino acid change/SNP selection criteria according to the change in amino acid polarity and charge.

We extracted the gynaecological cancer amino acid changes (*n* = 30) (Table 3) according to the selection criteria in Figure 5. 

### 2.2. Prediction of the Effects of R804C/R831C on SLC4A11 Protein Stability, Function and Physiochemical Properties

**Figure 6:**
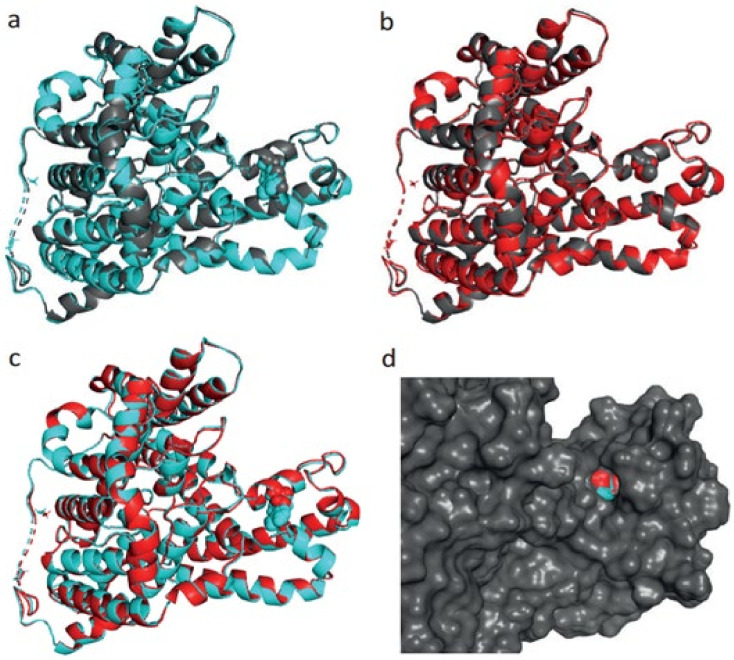
(**a**) Aligned structure of solute carrier family 4, sodium borate transporter, member 11 protein wildtype (918 aa, grey colour) and energy-minimised wildtype (cyan colour). (**b**) Aligned structure of SLC4A11 protein mutant (grey colour) and energy-minimised mutant (red colour). (**c**) Aligned structure of energy-minimised solute carrier family 4, sodium borate transporter, member 11 protein wildtype (cyan) and energy-minimised mutant (red). (**d**) Surface view of aligned structure of energy-minimised solute carrier family 4, sodium borate transporter, member 11 protein wildtype (cyan) and energy-minimised mutant (red).

Out of 30 gynaecological cancer amino acid changes, only 1 amino acid change, at R831C/R804C, has detected the structural damage of the protein *SLC4A11*, therefore, we modelled this protein (SLC4A11) with SNP at R831C/R804C in uterine corpus endometrioid carcinoma (Figure 6). The reason for the 2 different positions is due to the presence of 3 distinct N-terminal variants of human *SLC4A11*: 918 amino acid splice form 1 (where the mutation is at position 831), 891 amino acid splice form 2 (where the mutation is at position 804) and 875 amino acid splice form 3 (where the mutation is at position 788) [18,19].

For the 918 amino acid variant, the R831C substitution does not alter the secondary structure, but this substitution leads to the expansion of cavity volume by 97.2 Å^3^. Cavity also refers to a pocket on the surface (Figure 6). This substitution also results in a change between the buried and exposed state of the target variant residue. ARG is buried (RSA 7.6%) and CYS is exposed (RSA 20.7%). In the same protein, an increased z-score from −3.23 to −1.19 was noted, whereas for the mutant-type protein, the z-score changed from −3.24 to −1.16.

**Figure 7:**
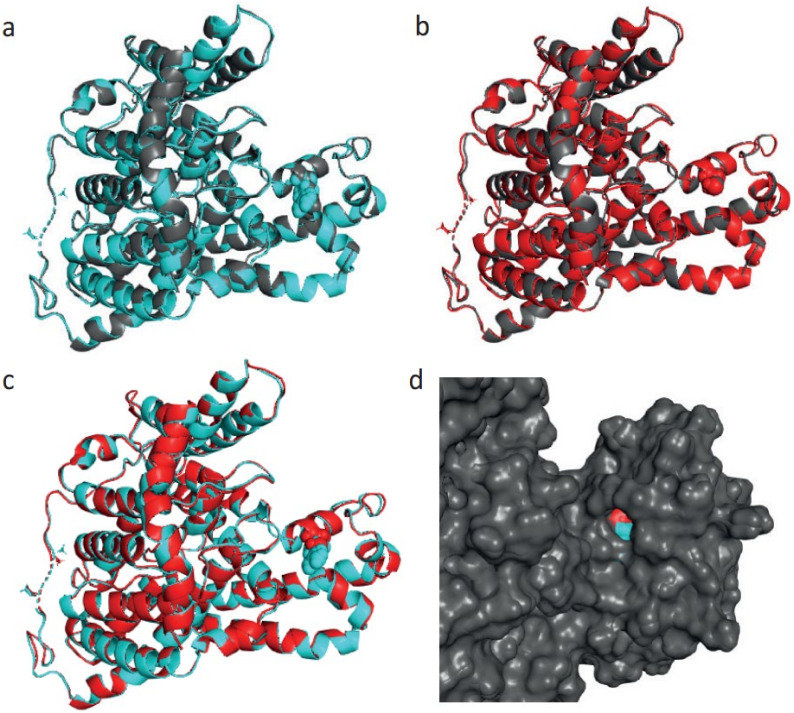
(**a**) Aligned structure of solute carrier family 4, sodium borate transporter, member 11 protein wildtype (891 aa, grey colour) and energy-minimised wildtype (cyan colour). (**b**) Aligned structure of SLC4A11 protein mutant type (grey colour) and energy-minimised mutant type (red colour). (**c**) Aligned structure of energy-minimised solute carrier family 4, sodium borate transporter, member 11 protein wildtype (cyan) and energy-minimised mutant type (red). (**d**) Surface view of aligned structure of energy-minimised SLC4A11 protein wildtype (cyan) and energy-minimised mutant type (red).

For the 891 amino acid variant, the R804C substitution does not alter the secondary structure, but this substitution leads to the expansion of cavity volume by 99.792 Å^3^. Cavity also refers to a pocket on the surface (Figure 7). This substitution also results in a change between the buried and exposed state of the target variant residue. ARG is buried (RSA 6.8%) and CYS is exposed (RSA 20.0%). Similarly, an increased z-score from −3.22 to −1.09 was also recorded for the wildtype protein and a similar change (from −3.22 to −1.11) for the mutant.

**Figure 8:**
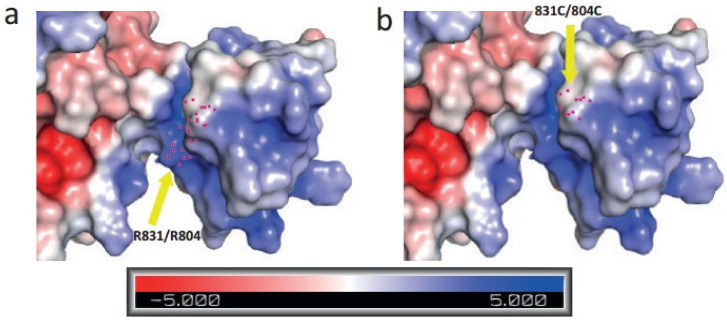
(**a**) An electrostatic potential surface of wildtype solute carrier family 4, sodium borate transporter, member 11 protein indicating amino acid residue ARG at position 831/804. (**b**) An electrostatic potential surface of mutant-type protein indicating amino acid residue CYS at position 831/804. In the colour legend, the red colour indicates negative potential, the blue colour indicates positive potential of the protein surface and the white regions correspond to fairly neutral potentials. Yellow arrow indicates towards the mutation site at position 831/804.

Moreover, we created an electrostatic potential surface for solute carrier family 4, sodium borate transporter, member 11 protein (Figure 8). As the colour legend indicates, the red colour (negative potential) arises from an excess of negative charges near the surface and the blue colour (positive potential) occurs when the surface is positively charged. The white regions correspond to fairly neutral potentials.

Arginine (R) is a positively charged, polar and hydrophilic amino acid in proteins that has a profound role in protein structure and function that involves electrostatic interactions and protein solvation [20]. Alternatively, cysteine (C) is a non-polar, uncharged and hydrophobic amino acid, and the substitution from R to C may have a deleterious impact on the protein hydration and electrostatic interactions of the protein. When we used PROVEAN (Protein Variation Effect Analyzer), a software tool which predicts whether an amino acid substitution has an impact on the biological function of a protein, it provided a score of −7.292 with the annotation “Deleterious” for both R831C and R804C. The default score threshold is currently set at −2.5 for binary classification (i.e., deleterious vs. neutral).

We have further evaluated changes in protein stability using MUpro: Prediction of Protein Stability Changes for Single-Site Mutations from Sequences [21,22], where Delta Delta G (DDG), a metric for predicting how a single point mutation will affect protein stability, was measured. In both variants, the predicted DDG was −0.704, suggesting a decrease in protein stability. Similar data were obtained from the BIOCOMP.UNIBO prediction server [23], with a DDG of −0.67 and a prediction of a disease-related mutation. Finally, we have used the DeepDDG server [24] that predicts the stability change of protein point mutations using neural networks and calculated a DDG value of −1.802 (kcal/mol).

## 3. Discussion

In this study, we provided a comprehensive overview of a wide repertoire of mutations of seven recently predicted biomarkers for OC that can be acquired using a number of in silico tools. These 7 genes present with 976 SNPs/mutations that are associated with human cancers, out of which 284 are related to female cancers that include ovarian, cervical, endometrial cancer, as well as endometrioid and uterine carcinomas. The most prevalent type of mutation occurring on six (i.e., *GBP5*, *IRS2*, *KRT4*, *MRPL55*, *RRS1* and *SLC4A11*) out of seven genes was missense mutation, followed by silent and 3′untranslated region (3′UTR) mutations. In the case of *LINC00707*, being a long non-coding RNA (lncRNA), non-coding transcript exon and intron mutations were the only two types identified in both all cancers and female ones. In both cases, *SLC4A11* had the largest percentage of mutations out of all 7 genes at 29.4% and 28.9%, respectively.

In missense mutations, there is a change of a single nucleotide, resulting in a codon that can produce a different amino acid. Using the Human Genome Database as a paradigm, it is evident that several missense mutations are linked with inherited predispositions to malignancies [25]. For example, in a recent analysis of more than 113,000 women, missense variants for *BRCA1, BRCA2* and *TP53* were associated with a risk of breast cancer [26]. Equally, a number of studies have indicated that mutations at the 3′UTR can drive oncogene activation or inactivation of tumour suppressors by altering the binding efficiency of microRNAs [27,28]. For example, a *GAPDH* mutation in the 3′UTR creates a miR-125b binding site, and as a result facilitates the development of OC [27].

On the other hand, the mutational landscape for the lncRNA *LINC00707* is quite different. We know that lncRNAs exhibit a complex biology and are involved in a number of processes, including gene transcription or gene silencing [29]. Although there is no published data on intronic mutations and their impact on *LINC00707*, a recent study highlighted their importance in cancer, since 64 tumour suppressors were affected by intronic mutations, and blood cancers showed higher proportions of deep intronic mutations [30].

We have then provided a deeper insight into the percentage of mutation of each of the seven genes of interest in all cancers and in female cancers. For the latter, the largest percentage (28.9%) was attributed to *SLC4A11*, with *GBP5* and *KRT4* exhibiting a high percentage as well (21.5% and 20.4%, respectively). In this cohort of cancers, the largest datasets were of uterine endometrioid carcinoma (*n* = 102) and uterine corpus endometrioid carcinoma (UCEC; *n* = 85). UCEC is the most common female pelvic malignancy, and the sixth most common gynaecological malignancy in females, with an estimated 417,367 new cases and 97,370 deaths worldwide in 2020 [31]. Despite the wide repertoire of therapeutic options for UCEC, there is an increase in the incidence of endometrial cancer. Of note, numerous shared and cancer type-specific mutation signatures have been identified, with UCEC depicting a number of clusters with distinct mutation frequencies [32]. Out of the seven genes in question, only one study associates the IRS2 polymorphism G1057D with endometrial cancer [33].

We then analysed the mutation impact on amino acid polarity, charge and water affinity, leading to the identification of 30 mutations in gynaecological cancers where amino acid changes lead to opposite polarity, charges and water affinity. Out of 30 gynaecological cancer amino acid changes, only missense mutation (i.e., R831C/R804C in UCEC) was suggestive of structural damage on the solute carrier family 4, sodium borate transporter, member 11 protein. Therefore, we modelled this protein and provided in silico evidence of how a change from arginine (R) to cysteine (C) can exert potential deleterious consequences.

*SLC4A11* is a member of the SLC4 family of bicarbonate transporters that is primarily expressed as an integral membrane protein, with aberrant expression in the cornea, thyroid, salivary gland and kidney. This transporter is also involved in sodium-mediated fluid transport in different tissues. The human *SLC4A11* gene encodes three splice variants at the NH2 terminus. These include the 918 variant A, the 891 amino acid variant B and the 875 amino acid variant C [18,19]. Of these, according to UniProt, SLC4A11-B is the canonical sequence. To date, most of the work on *SLC4A11* is concentrated on corneal dystrophies. Indeed, mutations of *SLC4A11* are the cause of congenital hereditary endothelial dystrophy (CHED) and some cases of late-onset Fuchs endothelial corneal dystrophy (FECD) [18]. Interestingly, one the mutations found in families with autosomal recessive corneal endothelial dystrophy (CHED2) was on arginine 804 (G804A). The authors of the study argued that the mutation can alter the hydrophobic interaction of methyl groups located in the arginine stem, thus impacting on the loop stability [34].

In this study, we have shown that (1) the R831C/R804C mutation is deleterious and (2) predicted ΔΔG values suggest that the mutation reduces the stability of the protein. As mentioned, DDG is the change in Gibbs free energy (Gibbs free energy (G) = Enthalpy (H) − Temperature (T) × Entropy (S)) [24]. There is also a strong structural explanation for the change in stability: Arg-831 is in a salt bridge with nearby Glu-519, so R831C will have a large enthalpic impact. However, we acknowledge that it is difficult to further dissect the functional impact of this change in stability without embarking on in vitro studies, mutating the protein in cellular models of UCEC. We also acknowledge that the cavity hypothesis is limited by the neglect of protein–membrane interactions in YASARA. Very recently, a new artificial intelligence system (AI) that predicts 3D protein structures with high accuracy has emerged, termed AlphaFold [35]. Subsequently, we have modelled our predicted structures of the two SLC4A11 protein variants with that of AlphaFold and there is 100% alignment in the R804 transmembrane region (Supplementary Figure S1), suggesting a conserved 3D configuration irrespective of the modelling software.

In terms of its role in female reproductive organs, the only data available come from a study in OC, where high expression of *SLC4A11* is a predictor for poor overall survival in serous OC (grade 3/4) [36]. Leveraging data from TCGA and GTEX, we also demonstrated significant upregulation of *SLC4A11* in UCEC (Supplementary Figure S2). Future studies should concentrate on gaining a deeper understanding of the actual role of this transporter protein in UCEC and how this deleterious mutation might affect its function, as the normal function(s) of *SLC4A11* in gynaecological malignancies still remains unclear.

## 4. Materials and Methods

### 4.1. Data Availability

Xena Repository: Somatic mutation data and sample phenotype information were extracted from the data generated by The Cancer Genome Atlas (TCGA) research network and TCGA somatic mutations (Pan-cancer Atlas), as published in the Xena repository hosted at the University of California Santa Cruz (UCSC) [37].

UK BioBank: Genetic variation/mutation data were extracted from PhenoScanner (version 2), which is a curated database holding publicly available results from large-scale genome-wide association studies (GWAS) for the UK Biobank data. This tool helps to facilitate “phenome scans”, the cross-referencing of genetic variants with a broad range of phenotypes, to help aid the understanding of disease pathways and biology.

cBioPortal: Genomic alterations across a set of patients were quarried from cBioPortal (for cancer genomics), an exploratory analysis tool for exploring large-scale cancer genomic datasets that hosts data from large consortium efforts, such as TCGA and TARGET, as well as publications from individual labs. The cBioPortal assists to explore specific genes or a pathway of interest in one or more cancer types.

Statistical Analysis: All unstructured data gathering, processing, modelling and statistical analyses were conducted using R (v. 4.1.0, The R Foundation for Statistical Computing, Vienna, Austria) under the R Studio desktop application (version 1.4.1717, RStudio, Boston, MA, USA).

### 4.2. Protein Structure Prediction Tools

UniProt Knowledgebase: The amino acid sequence of the protein of interest was extracted from the UniProt Knowledgebase (UniProtKB) (https://www.uniprot.org (accessed on 10 November 2021)), which is the central hub for the collection of functional information on proteins, with accurate, consistent and rich annotation. It records the information extracted from the literature and curator-evaluated computational analysis.

Protein Data Bank (RCSB PDB): We used the Protein Data Bank (PDB) (https://www.rcsb.org (accessed on 10 November 2021)) to gather the known protein structure information of our genes of interest. It is the single worldwide archive of structural data of biological macromolecules. It includes data obtained by X-ray crystallography and nuclear magnetic resonance (NMR) spectrometry submitted by biologists and biochemists from all over the world.

Phyre2: In order to predict the three-dimensional (3D) structure of our desired protein sequence/gene, we used Phyre2 (v. 2.0). The software assists with the construction of 3D models of our protein of interest based on the alignments between the hidden Markov model (HMM) of the desired sequence and the HMMs of known structure.

SWISS-MODEL: We also used a fully automated 3D protein structure homology-modelling server, SWISS-MODEL (https://swissmodel.expasy.org/ (accessed on 10 November 2021)), to predict the 3D structure of our desired protein sequence. Homology modelling is currently the most accurate method to generate reliable 3D protein structure models, as it makes use of experimental protein structures (“templates”) to build models for evolutionary-related proteins (“targets”).

AlphaFold: The Protein Structure Database (https://alphafold.ebi.ac.uk/ (accessed on 10 November 2021)), an AI system which is able to computationally predict protein structures with unprecedented accuracy and speed, was also used to predict the 3D structure.

Missense3D: Structural changes introduced by an amino acid substitution/SNP were measured and predicted by the Missense3D tool (http://missense3d.bc.ic.ac.uk/missense3d (accessed on 10 November 2021)).

YASARA Energy Minimisation Server: Energy minimisation of the protein was performed using the YASARA server (http://www.yasara.org/minimizationserver.htm (accessed on 10 November 2021)), and the YASARA application (v. 21.8.26) was used to view and save the 3D energy-minimised structure in PDB format.

PyMOL: Electrostatic potential surfaces, electron densities and three-dimensional (3D) visualisation of proteins were analysed by PyMOL (v. 2.4.1), which is an open-source molecular visualisation platform.

PROVEAN: Impacts on the biological function of protein sequence variations including single or multiple amino acid substitutions were predicted by the PROVEAN (Protein Variation Effect Analyzer) (v. 1.1) tool (http://provean.jcvi.org/ (accessed on 10 November 2021)) [38].

## Figures and Tables

**Table 1:** Data summary for the mutation samples from TCGA, UK BioBank and cBioPortal datasets. The “Total Samples” is with respect to the samples associated with the genes of interest.

Gene	Samples	TCGA	UK BioBank	cBioPortal
	Total Samples	713	950	647
	All cancers	713 (100%)	48 (100%)	647 (100%)
	Female cancers *	145 (20.33%)	7 (14.58%)	208 (32.14%)
***GBP5***	All cancers Female cancers	145 (20.33%) 27 (3.78%)	3 (6.25%) 1 (2.08%)	150 (23.18%) 54 (8.34%)
***IRS2***	All cancers Female cancers	114 (15.98%) 30 (4.20%)	8 (16.66%) -	82 (12.67%) 18 (2.78%)
***KRT4***	All cancers Female cancers	154 (21.59%) 22 (3.08%)	7 (14.58%) 2 (4.16%)	158 (24.42%) 50 (7.72%)
***LINC00707***	All cancers Female cancers	- -	24 (50%) 2 (4.16%)	- -
***MRPL55***	All cancers Female cancers	35 (4.90%) 10 (1.40%)	1 (2.08%) 1 (2.08%)	24 (3.70%) 9 (1.39%)
***RRS1***	All cancers Female cancers	57 (7.99%) 16 (2.24%)	1 (2.08%)-	38 (5.87%) 11 (1.70%)
***SLC4A11***	All cancers Female cancers	208 (29.17%) 40 (5.61%)	4 (8.33%) 1 (2.08%)	195 (30.13%) 67 (10.35%)

* Female cancers: ovarian, cervical/endocervical, uterine, breast and endometrial/uterine corpus endometrioid carcinoma.

**Table 2:** Data summary for the exon mutation samples used in this study from TCGA, UK BioBank and cBioPortal datasets to analyse the mutation impact at protein structure and function. Including amino acid polarity, charges and water affinity.

Feature	Count
**Exon Mutation**	**807 (100%)**
Non silent mutation	560 (69.39%)
Silent mutation	173 (21.43%)
Stop codon mutation	74 (9.16%)
**Amino Acid Polarity**	**560 (100%)**
Polar to Non-polar	104 (18.57%)
Non-polar to Polar	123 (21.96%)
No charge	333 (59.46%)
**Amino Acid Charge**	**560 (100%)**
Positive to Negative	1 (0.17%)
Positive to No charge	93 (16.60%)
No charge to Positive	37 (6.60%)
Negative to Positive	16 (2.85%)
Negative to No charge	31 (5.53%)
No charge to Negative	27 (4.82%)
No charge	355 (63.39%)
**Amino Acid Water Affinity**	**560 (100%)**
Hydrophobic to Hydrophilic	8 (1.42%)
Hydrophobic to Neutral	65 (11.60%)
Neutral to Hydrophobic	84 (15%)
Hydrophilic to Hydrophobic	47 (8.39%)
Hydrophilic to Neutral	76 (13.57%)
Neutral to Hydrophilic	46 (8.21%)
No charge	234 (41.78%)

**Table 3:** Data summary of the gynaecological cancer amino acid changes, where *n* = 30, showing opposite polarity, charges and water affinity. 1—USCS Xena and 2—cBioPortal.

Database	Gene	Cancer Type	Amino Acid Change	Mutation
1/2	*GBP5*	Cervical and Endocervical Cancer	R520I	Missense
1/2	*GBP5*	Uterine Corpus Endometrioid Carcinoma	R450W	Missense
1/2	*GBP5*	Uterine Corpus Endometrioid Carcinoma	R290C	Missense
1/2	*GBP5*	Uterine Corpus Endometrioid Carcinoma	P415H	Missense
2	*GBP5*	Uterine Endometrioid Carcinoma	R396W	Missense
2	*GBP5*	Uterine Endometrioid Carcinoma	F267C	Missense
2	*IRS2*	Uterine Endometrioid Carcinoma	E1150K	Missense
1/2	*KRT4*	Ovarian Serous Cystadenocarcinoma	R49P	5′UTR
1/2	*KRT4*	Cervical and Endocervical Cancer	E238K/E312K	Missense
1/2	*KRT4*	Uterine Corpus Endometrioid Carcinoma	R196M/R270M	Missense
1/2	*KRT4*	Cervical and Endocervical Cancer	R9P/R83P	Missense
1/2	*KRT4*	Uterine Corpus Endometrioid Carcinoma	R27I/R101I	Missense
2	*KRT4*	Uterine Endometrioid Carcinoma	E509K	Missense
2	*KRT4*	Uterine Endometrioid Carcinoma	G84D	Missense
2	*KRT4*	Uterine Endometrioid Carcinoma	D507V	Missense
2	*KRT4*	Uterine Endometrioid Carcinoma	R270M	Missense
2	*KRT4*	Uterine Endometrioid Carcinoma	G578D	Missense
2	*MRPL55*	Uterine Endometrioid Carcinoma	G20R	Missense
2	*MRPL55*	Uterine Endometrioid Carcinoma	R96C	Missense
2	*MRPL55*	Uterine Endometrioid Carcinoma	P86H	Missense
1/2	*RRS1*	Uterine Corpus Endometrioid Carcinoma	R83C	Missense
1/2	*RRS1*	Uterine Corpus Endometrioid Carcinoma	L157R	Missense
1/2	*SLC4A11*	Uterine Corpus Endometrioid Carcinoma	R831C/R804C	Missense
1/2	*SLC4A11*	Cervical and Endocervical Cancer	R309C/R282C	Missense
1	*SLC4A11*	Uterine Corpus Endometrioid Carcinoma	R50M	Missense
2	*SLC4A11*	Serous Ovarian Cancer	R488M	Missense
2	*SLC4A11*	Uterine Endometrioid Carcinoma	R629W	Missense
2	*SLC4A11*	Uterine Endometrioid Carcinoma	D149V	Missense
2	*SLC4A11*	Uterine Endometrioid Carcinoma	E562K	Missense
2	*SLC4A11*	Uterine Endometrioid Carcinoma	R157C	Missense

## Data Availability

Data can be available upon reasonable request.

## Supplementary Materials

The following supporting information can be downloaded at: https://www.mdpi.com/article/10.3390/ijms23031725/s1.
