# Supplementary material for: In Silico Study to Predict the Structural and Functional Consequences of SNPs on Biomarkers of Ovarian Cancer (OC) and BPA Exposure-Associated OC
Source: Int J Mol Sci. 2022 Feb 2;23(3):1725. doi: 10.3390/ijms23031725 (PMC8835975; doi:10.3390/ijms23031725)
Supplement: Supplementary file 1 [file ijms-23-01725-s001.zip › ijms-1538424-supplementary.pdf]

Supplementary Materials

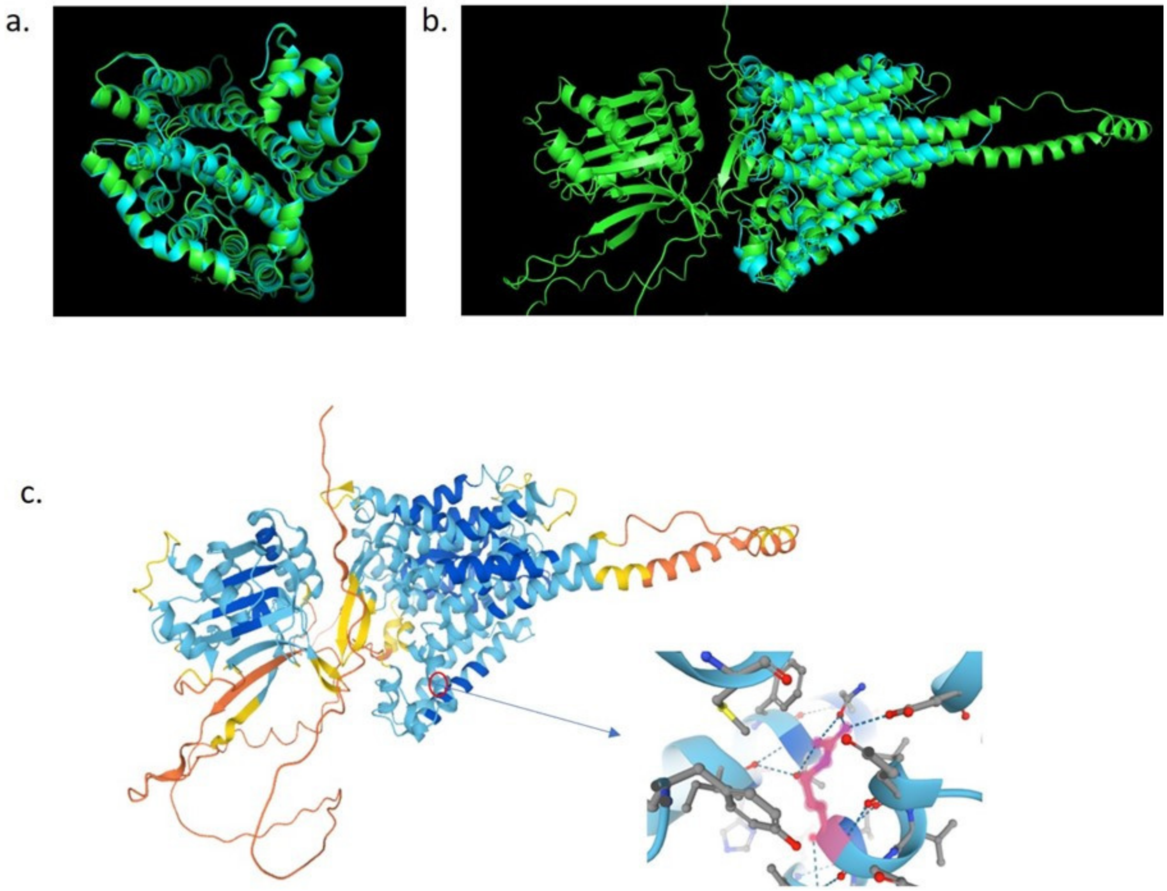

Supplementary Figure S1. Panel (**a**): Alignment of the 891 and 918 amino acid (aa) variants of SLC4A11. Panel (**b**): Alignment of 891aa based on Swiss Model (blue) with AlphaFold (green). Panel (**c**): Predicted structure of SLC4A11 from AlphaFold, with R804C (red circle; insert confidence score 89.89) demonstrating full alignment with previous predictions.

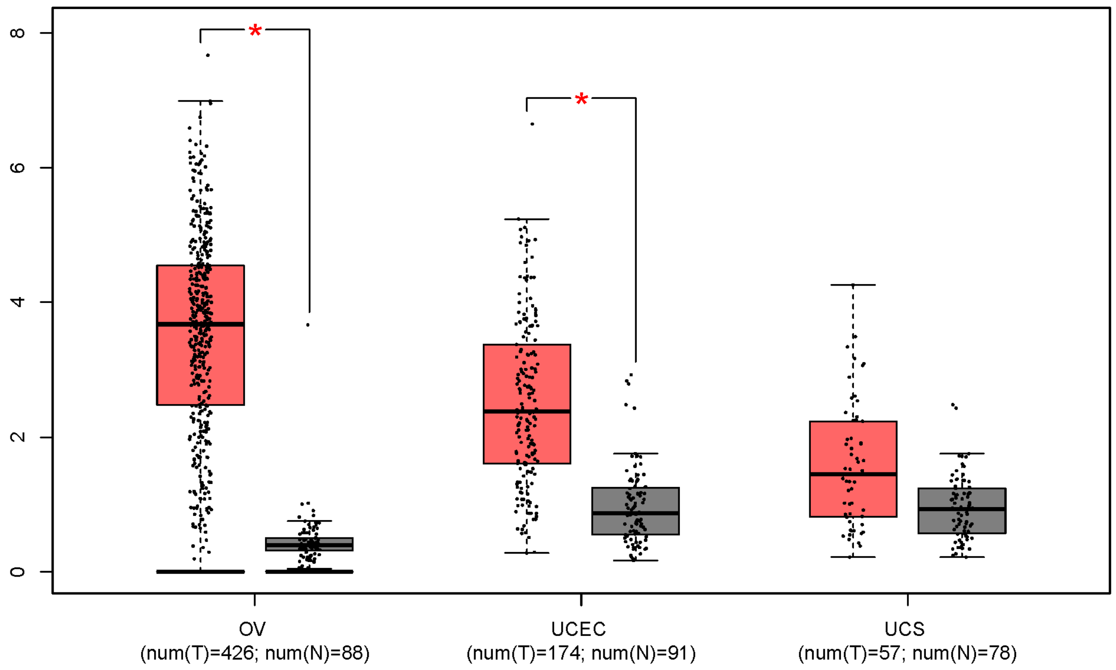

Supplementary Figure S2. Expression of SLC4A11 in ovarian cancer (OV), uterine corpus endometrial carcinoma (UCEC) and uterine carcinosarcoma (UCS). * p <0.05. T: tumour, N: normal, num: number of patients.
